# Assessment of urogenital schistosomiasis among basic school children in selected communities along major rivers in the central region of Ghana

**DOI:** 10.11604/pamj.2021.40.96.26708

**Published:** 2021-10-13

**Authors:** Evans Duah, Ernest Kenu, Edward Morkporkpor Adela, Hilda Adjoa Halm, Clement Agoni, Ransford Oduro Kumi

**Affiliations:** 1 Department of Epidemiology and Disease Control, School of Public Health, University of Ghana, Accra, Ghana,; 2 Clinical Laboratory, Cape Coast Teaching Hospital, Cape Coast, Ghana,; 3 Dream Laboratory Consult Limited, Cape Coast, Ghana,; 4 Tema Community 22 Polyclinic, Tema, Ghana

**Keywords:** Urogenital schistosomiasis, *Schistosoma haematobium*, school-age children, rivers, Ghana

## Abstract

**Introduction:**

urogenital schistosomiasis affects school-aged children with impacts on health, growth, and cognitive development. Basic schools along active water bodies have a possibility of a high infection among the children.

**Methods:**

we performed a school-based cross-sectional assessment of urogenital schistosomiasis among children in four selected rural communities along major rivers in the central region of Ghana. Three hundred and nine (309) basic school children class 1 to junior high school (JHS) 3 were recruited. Sociodemographic data and information on behavioral influences were collected with a structured written questionnaire. Laboratory examinations were conducted on fresh urine samples. Descriptive statistics and cross-tabulations with measures of association between variables, adjusted and unadjusted logistic regression analysis were performed on measured variables.

**Results:**

we recorded a 10.4% prevalence of urogenital schistosomiasis. Schools in communities along the Kakum river recorded the highest disease burden (65.6%). The odds of infection among pupils who engage in irrigation activities were 4 folds more than those who do not engage in irrigation activities (adjusted odds ratio (aOR) (95%CI): 4.3 (1.6-12.1), P-value=0.005). Pupils of caregivers who resort to self-medication using local herbal concoctions had 14-fold more odds of infection compared to those who visit the health facility (aOR (95%CI): 14.4 (1.4-143.1), P-value=0.006).

**Conclusion:**

poor health-seeking behaviors and lack of access to health facilities influenced the disease proportion among the children in these endemic communities.

## Introduction

Schistosomiasis is a neglected tropical disease [1] with an estimated 232 million people globally infected in 78 countries annually [2]. It is prevalent in sub-Saharan Africa, with 85% of the estimated number of global infections [3,4]. Studies in Ghana have recorded varying rates of urogenital schistosomiasis among different populations in Ghana using varying clinical diagnostics, markers and epidemiological indicators however, there is still an inadequate database in the District Health Information Systems (DHIMS) to generalize [5-11].

Children, women and people working in contact with freshwater bodies continue to be at risk [2,12]. Urogenital schistosomiasis is a major parasitic disease that affects school-aged children with impacts on health, growth, and cognitive development [7,13]. The World Health Organization´s (WHO) report shows that out of the 90 million global infections of schistosomiasis, 70.9 million of the cases were recorded among school-aged children in 2016 [14]. This influenced WHO´s roadmap to reach at least 75% of all school-age children in endemic areas by 2020 [14]. A study among school-aged children in two adjoining communities along the Kakum river in the central region of Ghana recorded prevalence rates of 27.5% and 17% (Apewosika and Putubiw respectively) [8]. The active play habits of children, their involvement in household, fishing, and farm irrigation activities increase their water contact hours which further increases their exposure to the infection [14,15]. In children, urogenital schistosomiasis presents with acute illnesses such as hematuria and anemia, however, fibrosis of the bladder and ureter, and subsequent kidney impairment may follow in chronic infections [16].

For years, clinicians and researchers have focused on presumptive diagnosis and case management [7,11,17-20]. Recently, inclusive approaches to better understand the dynamics in the transmission of the disease among populations have been employed [12]. Information on sociobehavioral influences such as health-seeking behaviors, the use of health facilities for schistosomal-related symptoms among others has proven to be a sustainable and effective approach to control and possibly eliminate urogenital schistosomiasis [21,22].

This study aimed to estimate the proportion of basic school children infected with urogenital schistosomiasis, to assess the sociodemographic and behavioral factors that influenced the infection rate in the selected communities. This further sought to guide a cost-effective immediate intervention program for the children. Ultimately, this study contributes to the World Health Organization´s policy direction and roadmap to control morbidity of schistosomiasis by 2020, along with its elimination as a public health problem by 2025 [23,24].

## Methods

**Study design:** this cross-sectional study was carried out among basic school children in four selected basic schools in 4 communities along 2 major rivers in the central region of Ghana. The study was carried out from 2^nd^ March 2020 to 31^st^ July 2020.

**Study location/area:** the study was conducted in 4 rural communities in 2 districts in the central region of Ghana. Sorodofo-Abaasa, Patasi, and Essiam are located in the Abura Asebu-Kwamankese District of the central region of Ghana whereas Effutu-Mampong is in the Cape Coast North District. The selected schools are situated along 2 major rivers in the region. These rivers are the Kakum River and the Srowie River. Two (2) communities each share a river. Sorodofo-Abaasa and Patasi share the Kakum river whilst Efutu-Mampong and Essiam share the Srowie river. Most indigenes in these communities are farmers and fisherfolks. These rivers are interconnected in tributaries. The Sorodofo-Abaasa basic and JHS, Patasi AME Zion School in the communities are 300 meters and 200 meters walking distance from the Kakum river respectively. However, the Essiam Catholic Basic and JHS and Effutu-Mampong basic and JHS are 100 meters and 150 meters walking distance from the Srowie river respectively. These communities essentially depend on the 2 rivers for their livelihoods including water for household chores, fishing, and irrigation activities. These communities and schools were selected based on the bio-ecologic distribution of *Schistosoma haematobium*.

**Study population***:* the study was conducted in 4 basic schools in the central region of Ghana. These were Sorodofo-Abaasa basic and JHS, Patasi AME Zion School, Essiam Catholic Basic and JHS, and Effutu-Mampong basic and JHS. The schools are located in Sorodofo-Abaasa, Patasi, Essiam, and Effutu-Mampong respectively. We conveniently recruited three hundred and nine (309) basic school children from class 1 to junior high school (JHS 3) who agreed to participate in the study.

**Sample size determination:**


$$n\geq \frac{P\left(1-P\right)Z^{2}{}_{\left(1-\alpha /2\right)}}{e^{2}}$$

where: n=sample size required; Z=Z score for 95% confidence interval = 1.96; P= 27.5% (Boye *et al*. 2016); e=margin of error (5%=0.05); using P=27.5%;


$$\begin{array}{l} n\geq \frac{0.275\left(1-0.0275\right){\left(1.96\right)}^{2}}{0.05^{2}}\\ n\geq 306. \end{array}$$

### Data collection

**Socio-behavioral information:** sociodemographic data and information on behavioral factors were collected with a structured written questionnaire. The questionnaire was explained and interpreted in the Fante local language to pupils taking into account content protection to ensure clarity in communication.

**Urine collection and transportation:** the school children were provided clean, dry, leak-proof, and wide-mouthed plastic urine sample containers. Fresh urine, 50 ml each, was collected between 10 am and 2 pm following the circadian pattern of ova excretion in urine and preferably, after a short physical exercise [11]. The samples were transported on a reverse cold chain (between 2-8°C on ice packs) for immediate laboratory examination within 2 hours of collection. This maintained the viability of the eggs and prevented the miracidia from hatching [25].

**Laboratory examination:** ten (10) ml of that same urine specimen was gently shaken and then centrifuged at 2000 rpm for 3-5 min, the supernatant discarded, wet mount made on a labeled frosted glass slide with a coverslip [11,19,26]. The wet deposit was examined via a light microscope using 10x and 40x objective lenses for *Schistosoma haematobium* eggs.

**Data analysis:** datasets were entered into Microsoft Excel 2013 and analyzed with Stata 16.0. Within-group frequencies were presented using descriptive statistics. The independent variables of statistical interest, the sociodemographic and behavioral factors, were modeled into a multivariate logistic regression analysis with *Schistosoma haematobium* positive infection as outcome variable. Results were presented in tables. P<0.05 was considered statistically significant in all analyses.

**Ethical considerations:** the study was approved by the Institutional Review Board (IRB) of the Noguchi Memorial Institute for Medical Research (NMIMR-IRB CPN 047/19-20). Written permission was sought from the regional education office and the headteachers of the schools. Parental consent was sought in writing and simple language was interpreted to their understanding. Child assent was sought from the basic school children in writing and simple language to their understanding.

## Results

**Figure 1:**
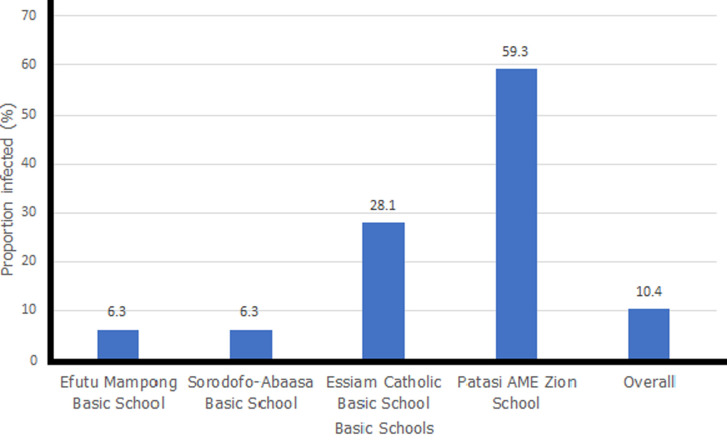
proportion of school children infected with *Schistosoma haematobium*

**General characteristics of the study population:** the overall proportion of the disease was recorded in 10.4% of the study population (Figure 1). However, Patasi AME Zion School recorded the highest burden of disease (59.3%).

**Factors associated with *Schistosoma haematobium* infection:** we assessed the knowledge, perception, and common practices among parents/guardians of pupils. Schools in communities along the Kakum river recorded the highest disease burden (65.6%) (Table 1). About 97.4% of the caregivers engage their wards to fetch water from the rivers for house chore activities and drinking (Table 2). About 60.8% engage their wards in fetching water from the rivers for farm irrigation. We assessed the predictors of *Schistosoma haematobium* infection among basic school pupils by awareness and practices of their caregivers (Table 3). Pupils who are engaged in irrigation activities are 4.3 folds more likely to be infected compared to pupils who are not engaged in irrigation activities (aOR, 95%CI: 4.3 (1.5-12.1), p-value=0.005). Pupils with no access to a health facility in their community are 6 folds more likely to be infected compared to those with access to a health facility (aOR, 95%CI: 6.3 (2.5-16.0), p-value=<0.001) (Table 3 (suite)). Pupils whose caregivers address their symptoms with self-medication using orthodox and local herbal concoctions are 14 folds more likely to be infected compared to those who visit the health facility (aOR, 95%CI: 14.3 (1.3-15.1), p-value=0.029) and (aOR, 95%CI: 14.4 (1.4-14.3), p-value=0.023) respectively.

**Table 1:** prevalence of *Schistosoma haematobium* infection

Variable	Total n (%)	SH infection	
		Positive	Negative
	N=309	N=32	N=277
Total	309	32 (10.4)	277 (89.6)
**River**			
Kakum river	140	21 (65.6)	119 (43.0)
Srowie river	169	11 (34.4)	158 (57.0)

Percentages: within group; SH: Schistosoma haematobium

**Table 2:** knowledge, perception and common practices among parents/guardians of pupils

Variable	Total n (%)	Efutu Mampong Basic School N=71	Sorodofo-Abaasa Basic School	Essiam Catholic Basic School	Patasi AME Zion School
	**N=309**	**N=71**	**N=67**	**N=98**	**N=73**
**Knowledge**					
Yes	256 (82.9)	58 (81.7)	64 (95.5)	73 (74.5)	61 (83.6)
No	53 (17.1)	13 (18.3)	3 (4.5)	25 (25.5)	12 (16.4)
**Awareness of child’s signs/symptoms**					
Yes	112 (36.3)	14 (19.7)	30 (44.8)	28 (28.6)	40 (54.8)
No	197 (63.7)	57 (80.3)	37 (55.2)	70 (71.4)	33 (45.2)
**Perception**					
Parasite	207 (67.0)	50 (70.4)	63 (94.0)	53 (54.1)	41 (56.2)
Spiritual	31 (10.0)	8 (11.3)	1 (1.5)	20 (20.4)	2 (2.7)
Water	12 (3.9)	0 (0.0)	0 (0.0)	0 (0.0)	12 (16.4)
No idea	59 (19.1)	13 (18.3)	3 (4.5)	25 (25.5)	18 (24.7)
Engage children in activities (multiple response) *					
Fishing	61 (19.7)	5 (7.0)	5 (7.5)	31 (31.6)	20 (27.4)
Irrigation	188 (60.8)	31 (43.7)	26 (38.8)	71 (72.5)	60 (82.2)
Drinking/house chores	301 (97.4)	64 (90.1)	66 (98.5)	98 (100.00)	73 (100.0)
None	5 (1.6)	5 (7.0)	0 (0.0)	0 (0.0)	0 (0.0)

* Multiple responses

**Table 3:** factors associated with *Schistosoma haematobium* infection

		Unadjusted logistic regression		Adjusted logistic regression	
Variable	n (%)	OR (95% CI)	P-value	aOR (95% CI)	P-value
**Awareness of child's signs/symptoms**					
Yes	112 (36.3)	1		1	
No	197 (63.7)	0.611 (0.292-1.276)	0.190	0.432 (0.182-1.025)	0.057
**Engage children in activities**					
**Fishing**					
Engaged	61 (19.7)	0.246 (0.572-1.060)	0.060	0.174 (0.037-0.809)	0.026*
Unengaged	248 (80.3)	1		1	
**Irrigation**					
Engaged	188 (60.8)	3.076 (1.227-7.713)	0.017*	4.349 (1.559-12.131)	0.005*
Unengaged	121 (39.2)	1		1	

Percentages: within group

**Table 3(suite):** factors associated with *Schistosoma haematobium* infection

		Unadjusted logistic regression		Adjusted logistic regression	
Variable	n (%)	OR (95% CI)	P-value	aOR (95% CI)	P-value
**Access to a health facility**					
Yes	236 (76.4)	1		1	
No	73 (23.6)	6.036 (2.807-12.977)	0.001*	6.344 (2.515-16.003)	<0.001*
**Payment plan**					
NHIS	105 (33.9)	1		1	
Self	204 (66.1)	1.617 (0.699-3.735)	0.261	1.219 (0.439-2.389)	0.704
**First intervention for the child's symptom**					
Health facility	48 (15.5)	1		1	
Self-medication (orthodox)	19 (6.2)	12.533 (1.298-12.962)	0.029*	14.319 (1.304-15.115)	0.029*
Self-medication (herbs)	26 (8.4)	20.889 (2.436-17.090)	0.006*	14.357 (1.440-14.315)	0.023*
Nothing	19 (6.2)	5.529 (0.470-6.962)	0.174	6.393 (0.453-9.127)	0.169
**The tendency to take your ward to a health facility**					
When the child is not well	219 (70.9)	1		1	
Haematuria	13 (4.2)	0.786 (0.097-6.346)	0.821	1.706 (0.160-18.089)	0.658
When child is dying	77 (24.9)	1.407 (0.631-3.139)	0.404	0.856 (0.303-2.302)	0.729
**Last visit to health facility (months)**					
<6	76 (24.6)	1		1	
6-12	117 (37.9)	2.088 (0.726-6.006)	0.172	1.365 (0.399-4.671)	0.620
>12	116 (37.5)	1.638 (0.553-4.853)	0.373	0.585 (0.156-2.188)	0.426

Percentages: within group

## Discussion

This study aimed to estimate the proportion of basic school children infected with urogenital schistosomiasis, to assess the sociodemographic and behavioral factors that influenced the infection rate in the selected communities. We recorded a 10.4% prevalence of urogenital schistosomiasis. Schools in communities along the Kakum river recorded the highest disease burden (65.6%). The odds of infection among pupils who engage in irrigation activities were 4 folds more than those who do not engage in irrigation activities (aOR, (95%CI): 4.3(1.6-12.1), P-value=0.005). Pupils of caregivers who resort to self-medication using local herbal concoctions had 14-fold more odds of infection compared to those who visit the health facility (aOR (95%CI): 14.4 (1.4-143.1), P-value=0.006).

The fight against urogenital schistosomiasis in Ghana has met varying phases of successes and failures. The current study assessed the socio-demographic and socio-behavioral influences on the burden of *Schistosoma haematobium* infection in the central region of Ghana. This concept sought to provide a sustainable and effective approach to control urogenital schistosomiasis, identify some possible factors leading to the inefficiencies in the control and elimination of the disease among school-aged children in the region. Though the current study recorded a relatively high proportion of children with urogenital schistosomiasis, the study communities were hypo-endemic areas compared with similar studies. The reported burden of 10.4% challenges similar studies in hyper-endemic areas in Western Kenya and South-Western Nigeria [20,27]. These settings recorded 37.6% and 78.1% prevalence of *Schistosoma haematobium* infection respectively. The seemingly low burden of infection compared to that of Western Kenya´s [27] may be attributed to the higher level of activeness or water contact hours of the respondents in their studies.

However, this burden agrees with a cross-sectional study among children in Niakaramandougou, Côte d´Ivoire [28]. In their study, they estimated the prevalence and intensity of the infection among children in the community as impacted by a rice irrigation farm cultivation. They recorded *Schistosoma haematobium* prevalence of 10%. The common irrigation farming culture shared by these communities cannot be overlooked hence the agreement with the current study. A similar study was performed among children in Putubiw and Apewosika communities along the Kakum river in 2016 recorded a prevalence of 20.8% [8]. Over the years, several measures have been put in place by stakeholders to address the findings. Annual mass drug administration (MDA) programs by the Ghana Health Service and the Ghana Education Service, education programs by teachers and traditional leaders, NGOs in school durbars may have contributed to this decline we have reported in the current study. However, if the children continue to live under the same environmental conditions and insist on the same behavior, then *Schistosoma haematobium* elimination may never be achieved in this aspect of the region. The continuous active play habits of children and caregivers engaging their wards in water contact activities such as fetching water for house chores and irrigation enhance re-infections. These behaviors stall disease eradication programs and interventions. About 97.4% of the caregivers engage their wards to fetch water from the rivers for house chore activities and drinking (Table 2).

About 60.8% engage their wards in fetching water from the rivers for farm irrigation. To model the activities along with the water bodies against the disease outcome, pupils who are engaged in irrigation activities are 4.3 folds more likely to be infected compared to pupils who are not engaged in irrigation activities. A case study in the Nile delta of Egypt significantly associated irrigation farming with urogenital schistosomiasis [29]. This is also in consonance with an irrigation system report on 46 African countries in 2006 [30]. The report indicated that most irrigation systems in Africa create favorable conditions for inhabitation and breeding of intermediate hosts hence subsequent transmission of schistosomiasis. This is common in farmers who practice surface irrigation. Clearly, in an indigenous community setting, older caregivers rely on their wards to do house chores and farming activities. This is embedded in African ethics, culture, values, and morals for the young to serve the old [31]. These activities hence expose the younger ones to the infection. This explains the increased infection among children who are engaged in such chores.

The schools along the Kakum river recorded the highest disease burden and this was reported in the Patasi AME Zion School. This is actively associated with the absence of a health facility in the community. In the current report, a lack of access to a health facility was associated with increased odds of infection. Health facilities serve as safe havens for communities in most settings. In the current study, only 15.5% of the caregivers report to health facilities. The remaining resort to alternatives such as self-medications and spiritual healers whilst others prefer to do nothing. This agrees with a questionnaire-based cross-country survey in Ghana in 2010 [21]. They reported that most communities in the local communities depend on allopathic self-medication as an alternative action. In Coastal Kenya, self-medication was the first intervention [32]. About 2% of infected people resorted to local herbs whereas 59.9% used antimalarial drugs for their *Schistosoma haematobium* infection symptoms. Undoubtedly, this action can be further attributed to the inadequate health facilities in the local communities. It is however not surprising to find several health facilities crowded in the cities of Ghana with inadequate for the local communities and villages.

The ability to afford health care influenced their likelihood to be infected with *Schistosoma haematobium*. The roll-out of the National Health Insurance Scheme as a social intervention program by the Government of Ghana was to provide financial access to quality health care for residents in Ghana, yet some marginalized groups have still not been able to subscribe to the service and 6% to 18% of the insured households still make catastrophic payments out of pocket [33]. These incidents influence their health-seeking behaviors and decisions to take action for their wards´ symptoms. The continuous demand for out-of-pocket payments from the community members influences their reluctance to visit the health facilities. This confirmed the revelation that 37.5% of the caregivers ever visited the health facility more than a year ago. In 2010, a survey concluded that the perceived severity of the disease is the most important determinant of seeking health care or visiting a health facility in Ghana [21]. Whilst others depend on the annual mass drug administration (MDA) for their wards, coverage has always been a challenge.

Generally, the study highlights the need to factor in behavioral assessment and the use of health facilities in community and school-based disease control programs. However, the current study failed to evaluate possible environmental and geographical influences in a more comprehensive epidemiological assessment of urogenital schistosomiasis among the basic school children in the local communities.

## Conclusion

Urogenital schistosomiasis was common among the school-age children in the selected communities. Caregivers who paid attention to the well-being of their wards, do not engage them in activities that expose them to infection as well as engaged in intermittent medical examinations, had reduced odds of been infected. Sociobehavioral change and health equity are therefore crucial in achieving WHO´s roadmap to reach at least 75% of all school-age children in endemic areas by 2020.

### What is known about this topic

- Urogenital schistosomiasis is a major parasitic disease affecting school-age children with impacts on health, growth, and cognitive development;
- The World Health Organization´s (WHO) report shows that out of the 90 million global infections of schistosomiasis, 70.9 million of the cases were recorded among school-aged children in 2016.

### What this study adds

- This study assessed and estimated the disease burden in the selected communities to guide a cost-effective immediate intervention program for the children;
- The study demonstrated that the sociobehavioral activities of the school-age children and their caregivers influence the transmission and distribution of urogenital schistosomiasis among the school-age children in the selected communities.
